# Navy Bean Supplementation in Established High-Fat Diet-Induced Obesity Attenuates the Severity of the Obese Inflammatory Phenotype

**DOI:** 10.3390/nu13030757

**Published:** 2021-02-26

**Authors:** Jennifer M. Monk, Wenqing Wu, Dion Lepp, K. Peter Pauls, Lindsay E. Robinson, Krista A. Power

**Affiliations:** 1 Department of Human Health and Nutritional Sciences, University of Guelph, Guelph, ON N1G 2W1, Canada; jmonk02@uoguelph.ca (J.M.M.); lrobinso@uoguelph.ca (L.E.R.); 2 Guelph Research and Development Centre, Agriculture and Agri-Food Canada, Guelph, ON N1G 5C9, Canada; wenqing.wu@canada.ca (W.W.); Dion.Lepp@canada.ca (D.L.); 3 Department of Plant Agriculture, University of Guelph, Guelph, ON N1G 2W1, Canada; ppauls@uoguelph.ca; 4 School of Nutrition Sciences, University of Ottawa, Ottawa, ON K1H 8L1, Canada

**Keywords:** obesity, navy beans, caloric restriction, intestinal health, epithelial barrier, adipose tissue inflammation

## Abstract

Cooked common beans (*Phaseolus vulgaris*) improve intestinal health in lean mice and attenuate intestinal dysbiosis and inflammation when consumed concurrent with obesity development. We determined the effects of a high-fat (HF) bean supplemented diet in mice with established obesity (induced by 12 weeks of HF diet (60% fat as kcal)) compared to obese mice consuming a HF or low-fat (LF) weight loss control diet. Obese C57BL/6 male mice remained consuming HF for eight weeks or were randomly switched from HF to an isocaloric HF with 15.7% cooked navy bean powder diet (HF→HFB) or LF (11% fat as kcal; HF→LF) (*n* = 12/group). HF→HFB improved the obese phenotype, including (i) fecal microbiome (increased *Prevotella*, *Akkermansia muciniphila*, and short-chain fatty acid levels), (ii) intestinal health (increased *ZO-1*, *claudin-2*, *Muc2*, *Relmβ*, and *Reg3γ* expression), and (iii) reduced adipose tissue (AT) inflammatory proteins (NFκBp65, STAT3, IL-6, MCP-1, and MIP-1α), versus HF (*p* < 0.05). Conversely, HF→LF reduced body weight and circulating hormones (leptin, resistin, and PAI-1) versus HF and HF→HFB (*p* < 0.05); however, AT inflammation and intestinal health markers were not improved to the same degree as HF→HFB (*p* < 0.05). Despite remaining on a HF obesogenic diet, introducing beans in established obesity improved the obese phenotype (intestinal health and adipose inflammation) more substantially than weight loss alone.

## 1. Introduction

Obesity is a chronic disease [1] characterized by low-grade systemic and adipose tissue (AT) inflammation (e.g., resistin, leptin, tumor necrosis factor alpha (TNFα), interleukin (IL)-6), metabolic disorders (e.g., hyperglycemia, insulin resistance, and dyslipidemia), and a dysbiotic and dysfunctional intestinal microenvironment [2], which collectively drive comorbidities, including type 2 diabetes, cardiovascular diseases (CVD), and cancer [3,4]. A compromised intestinal microenvironment contributes to an impaired intestinal epithelial barrier (i.e., increased permeability) [5,6] and metabolic endotoxemia, which can stimulate AT and systemic inflammation and metabolic dysfunction [6,7,8,9]. Therefore, targeting the intestinal microenvironment to attenuate obesity-associated inflammation and metabolic dysfunction may be a promising intervention strategy.

Fermentable nondigestible carbohydrates (NDCs), such as soluble fiber, resistant starch, and galacto- and fructooligosaccharides, as well as polyphenolic phytochemicals, have been shown to beneficially modulate the microbiome, improve intestinal barrier function and integrity, and attenuate inflammation and metabolic abnormalities associated with obesity [10,11,12,13,14]. Plant-based whole foods are enriched in fermentable NDCs and an array of polyphenolic compounds and are readily available in favor of consuming purified nutritional supplements. Previously, we have shown that dietary pulses (e.g., beans, chickpeas, and lentils) can improve intestinal health by modulating the cecal and/or fecal microbial composition and activity and by improving the mucus and epithelial barrier integrity and function in lean mice [15,16,17,18,19]. Many of these beneficial effects of beans on intestinal health may be driven by microbial-derived metabolites, including fermentation of NDCs enriched in beans (including soluble fiber, resistant starch, and galacto-oligosaccharides) as well as phenolic compounds, which are the dietary precursors for intestinal health-promoting metabolites (short-chain fatty acids (SCFAs), acetic acid, propionic acid and butyric acid [20,21], and secondary phenolic metabolites [22,23,24]).

The effect of bean or pulse food supplementation in individuals living overweight and/or with obesity is commonly combined with caloric restriction, and within this context, the addition of pulses exerts a modest improvement on body weight and/or body mass index (BMI; reviewed in [25]). Similarly, a meta-analysis on the effect of pulse consumption on body weight, waist circumference, and body fat when combined with either a caloric restriction or weight maintenance diet showed it was associated with very modest weight loss (i.e., −0.29 and −1.74 kg, respectively) [26], thereby indicating that the beneficial effects of pulses, such as beans, in obesity would likely influence other elements of the obese phenotype besides weight loss, including glycemia and dyslipidaemia (reviewed elsewhere ([27,28,29]). The impact of bean consumption has been investigated more thoroughly in rodent models of obesity [30,31,32,33], in which pulses supplemented into a high-fat (HF) diet can attenuate the negative consequences of elevated dietary fat on intestinal health, metabolic abnormalities, and/or inflammation. For example, in obesity-sensitive rats, diet supplementation with 60% cooked and dried beans for 26–29 days resulted in reduced fat mass and serum triglyceride concentrations [31], while a seven-week high-fat diet supplemented with cooked red beans (30% *wt/wt*) lowered the circulating leptin and cholesterol concentrations [33]. Recently, we showed that a high-fat diet supplemented with 15% (*wt/wt*) cooked navy beans consumed throughout the development of obesity attenuated the resultant obese phenotype by altering the dysbiotic obese fecal microbiota community structure and reducing intestinal epithelial barrier permeability, visceral AT inflammation, and metabolic dysfunction [32]. The results of that work are also supported by a recent study demonstrating that 40% cooked bean supplementation into a high-fat diet during obesity development can improve aspects of intestinal health and reduce adiposity compared to high-fat control mice [30]. What is unknown is the impact of introducing beans into a high-fat diet once the obese phenotype has already been established. Therefore, the objective of the current study was to determine the effect of cooked navy bean supplementation in established obesity on critical aspects of intestinal and metabolic health and AT inflammation.

## 2. Materials and Methods

### 2.1. Experimental Design and Diets

Navy beans (ACUG 10-B2 cultivar; provided by the University of Guelph Bean Breeding program) were cooked, freeze-dried, and powdered as we had described previously [18,32,34]; the proximate analysis has been published previously [32]. Experimental procedures were approved by the animal care committee (University of Guelph; animal use protocol #3115) in accordance with the guidelines of the Canadian Council of Animal Care. A total of 36 C57BL/6 male mice (4 weeks old) purchased from Charles River (Portage, MI, USA) were housed 3 mice/cage as previously described [18,34]. Mice were acclimatized to the low-fat (LF) basal diet for 1 week prior to consuming the HF diet for 12 weeks to establish the obese phenotype [32]. Subsequently, obese mice were assigned to one of three experimental diets, such that the average body weight (BW)/group were similar, for 8 weeks of dietary intervention (*n* = 12/dietary group): (i) HF: remained on the HF diet, (ii) HF→HFB: introduction of beans in established obesity via switching from the HF to the isocaloric high-fat bean (HFB) diet supplemented with 15.7% (*wt/wt*) cooked navy bean powder, or (iii) HF→LF: weight loss controls induced via switching from the HF to the LF diet.

The caloric densities of the two high-fat diets were similar (18% protein, 20% carbohydrate, 59% fat, and 2.7% fiber by kcal) (Table 1), as was the total fiber content (7% *wt/wt*). However the fiber profile differed; the HF contained insoluble cellulose, whereas the HF→HFB diet comprised a mixture of insoluble and soluble fibers. The supplementation level of the bean powder (15.7% *wt/wt*) was selected to represent a level of pulse intake in humans similar to 1 cup/day [35,36,37]. The LF diet consisted of 11% fat, 59% carbohydrate, 26% and protein (as kcal) [32]. All diets were prepared by Teklad, Envigo, USA.

Diet intake and BW were measured twice/week throughout the dietary intervention period. At the end of the dietary intervention period (12 weeks to establish the obese phenotype plus 8 weeks of dietary intervention in established obesity), freshly expelled fecal pellets were collected from individual mice and placed in sterile containers (<2 min). Then, fecal pellets were submerged in liquid nitrogen and stored at −80 °C for later analyses (microbial community structure by 16S rRNA gene sequencing or SCFA concentrations by gas chromatography (GC)).

### 2.2. Serum Adipokines and Lipopolysaccharide Binding Protein (LBP) Concentrations

Blood was collected by cardiac puncture at euthanasia. Serum concentrations of leptin, resistin, plasminogen activator inhibitor-1 (PAI-1), and insulin were measured using a diabetes-plex multiplex assay (Bio-Rad, Mississauga, ON, Canada) as per the manufacturer’s instructions. Serum adiponectin concentrations were measured individually as a single-plex assay (Bio-Rad). All multiplex assays utilized the Bio-Plex 200 System and the accompanying software, Bio-Plex Manager version 6.0 (Bio-Rad). Serum concentrations of LBP were analyzed using a Hycult^®^ Mouse LBP ELISA Kit (PA, USA, #HK205) as per manufacturer’s instructions. Serum was diluted 1000× in assay buffer prior to analysis. Absorbance was measured at 450 nm using a PowerWave XS2 plate reader and Gen5 microplate data collection and analysis software version 1.11 (BioTek, VT, USA, PN#MQX200R2), and results were expressed as ng/mL.

### 2.3. Adipose and Intestinal Tissue Collection

Visceral AT (epididymal fat depot) was excised and weighed. Intact colon tissue was collected from the cecocolonic junction to the rectum. Tissues were weighed and snap-frozen in liquid nitrogen and stored at −80 °C to await further analyses.

### 2.4. Colon mRNA Expression

Proximal colon RNA was extracted and purified using the RNA/Protein Purification Plus Kit (Norgen Biotek, Thorold, ON, Canada). cDNA was generated from 2 µg of total RNA using the High-Capacity cDNA Reverse Transcription Kit (Applied Biosystems, Foster City, CA, USA), and qRT- PCR analyses were conducted using Power SYBR Green PCR Master Mix (Applied Biosystems) as described previously [15,32]. The data was analyzed using the ∆∆CT method with data normalized to the level of expression of the *Rplp0* housekeeping gene [15,32]. Primer sequences have been validated and published previously [15,16,18,32,38,39,40].

### 2.5. Transcription Factor Activation, Cytokine, and Chemokine Protein Expression in Epididymal AT

AT was homogenized at 3500 rpm (Powerlyser, Mo Bio Laboratories, Carlsbad, CA, USA) in 1 mL of RIPA buffer supplemented with 1 mM PMSF and 1X protease inhibitor cocktail (Cell Signaling Technology, Danvers, MA, USA) as described in [32]. Epididymal AT protein (25 μg) was utilized to determine the activation level of the inflammatory transcription factors NFκB p65 and STAT3 by measuring the ratio of phosphorylated to total protein by InstantOne ELISA as per the manufacturer’s instructions (Invitrogen/Fisher Scientific, Burlington, ON, Canada) for total NFκB p65, phosphorylated-NFκB p65 (Ser 536), total STAT3, and phosphorylated-STAT3 (Tyr 705). Epididymal AT cytokine and chemokine protein expression (TNFα, IL-6, MCP-1, MIP-1α, and MIP-1β) was measured by multiplex (Bio-Rad) utilizing 25 μg of protein/sample.

### 2.6. Fecal 16S rRNA Gene Sequencing

The QiaAmp DNA Stool Mini Kit (Qiagen, Valencia, CA, USA) was used to extract genomic DNA from the collected fecal pellets. Sequencing libraries of the 16S V3-4 region were prepared according to the Illumina 16S Metagenomic Sequencing Library Preparation Guide [16,18,32,38], and 16S rRNA gene sequencing was performed exactly as described previously [16,18,32,38].

### 2.7. Sequence Processing and Diversity Analysis

Microbiota diversity analysis was performed with QIIME 2 (2019.7.0) [41]. Briefly, 300 bp paired-end reads were processed with DADA2 [42] to denoise, remove chimeric sequences and singletons, join paired-ends, and dereplicate sequences to produce unique amplicon sequence variants (ASVs). Taxonomic classification of the resulting feature table was performed with VSEARCH [43] and the Greengenes 99% OTU sequences [44] as reference. ASVs were discarded if they had fewer than 10 instances across all samples, were present in fewer than two samples, or were not assigned taxonomy at the phylum level. Multiple sequence alignment of ASV representative sequences was performed with MAFFT [45], and a rooted phylogenetic tree was constructed with FastTree [46]. Core diversity analysis was performed using a sampling depth of 8000 sequences to plot taxonomic relative abundances, calculate alpha-diversity metrics (Chao1 (species richness), Shannon (species evenness and diversity), and Pielou’s evenness), and to generate dissimilarity matrices based on Bray–Curtis, Jaccard, and UniFrac distances, which were used for principal component analyses (PCoA) [47]. PERMANOVA analysis was used to determine β-diversity distance matrix differences between dietary groups. Significant differences (*p* < 0.05) in taxa abundance between groups were determined by the Kruskal–Wallis test, followed by Dunn’s multiple comparison test. The *p*-values were adjusted for multiple testing using the Benjamin, Kreiger, and Yekutieli method.

### 2.8. Fecal SCFA Analyses

Fecal SCFA concentrations (acetate, propionate, and butyrate) were measured by GC as previously described [15,34,38]. In brief, fecal samples (~50 mg) were freeze-dried in a Freezone 12 bulk tray dryer (Labconco, Canada), the moisture content was determined, and the samples were then homogenized in MilliQ water (Ultrapure water system; Barnstead International, Dubuque, IA, USA) to obtain a 10% (*w*/*v*) fecal solution. The suspension was centrifuged for 10 min at 10,000× *g* (rcf), and the pH was measured (Thermo Scientific™ Orion Star™ A111 pH Benchtop Meter, Thermo Scientific ROSS MICRO PH ELECTRODE, Canada). Then, 0.5 M 2-ethylbutyric acid (Aldrich, #109959) in formic acid was added to the supernatant. The supernatant was filtered (0.2 μm PVDF Syringe Filter; Chromatographic Specialties) and injected (1 μL) in triplicate into the GC (Agilent 6890, Canada), equipped with a flame ionization detector and a Nukol Capillary GC Column (60 m × 0.25 mm × 0.25 μm, Sigma-24108 SUPELCO). Helium was used as the carrier gas. The initial oven temperature was 100 °C and was increased to 200 °C at a rate of 10 °C/min; the injector and detector temperatures were maintained at 200 and 250 °C, respectively. The total running time was 20 min for each injection. The peaks were identified by comparing their retention times with Volatile Acid Standard Mix (Sigma, #46975-U). The data was managed using HPCHEM software (Agilent Technologies, Canada), and fecal SCFA concentrations were expressed as μmol/g of dry fecal weight.

### 2.9. Statistics

Food intake and BW changes over time were assessed by repeated measures two-way ANOVA (main effects: diet group and day). One-way ANOVA (main effect: diet) and Student–Newman–Keuls (SNK) or Tukey’s multiple comparison test was used for post-hoc analyses for assessment of significant difference between dietary groups (*p* < 0.05) for all other outcomes using GraphPad Prism 8.0 (GraphPad Software, Inc., La Jolla, CA, USA). All data are expressed as mean ± standard error.

## 3. Results

### 3.1. Changes in BW, Energy Intake, and Metabolic Health

**Figure 1:**
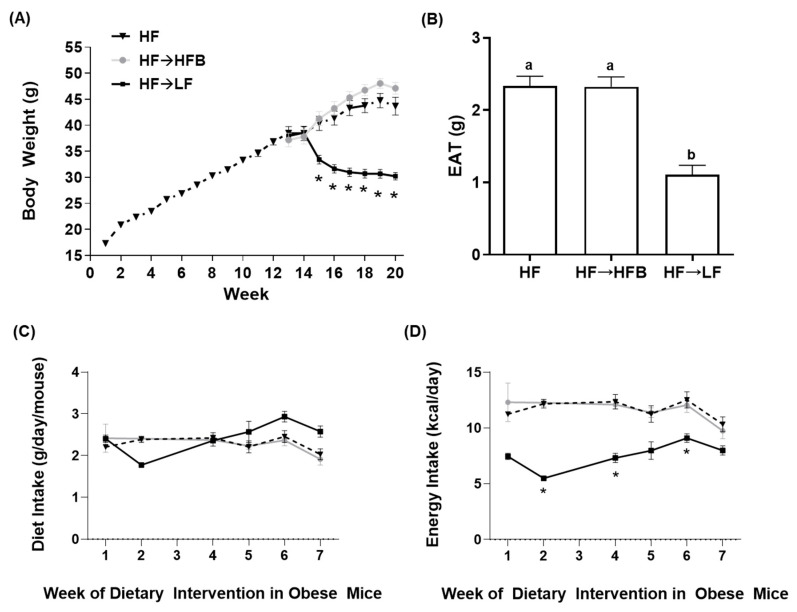
Body weight (**A**), epididymal adipose tissue (EAT) weight (g) (**B**), diet intake (g/day) (**C**), and energy intake (kcal/day) (**D**). Values are mean ± SEM; *n* = 12/dietary group. All mice were fed the high-fat (HF) diet for 12 weeks to establish the obese phenotype prior to eight weeks of dietary intervention (weeks 12–20) consisting of the HF diet (black triangles, dotted line), the HF diet switched to the isocaloric high-fat bean (HFB) diet (HF→HFB; gray squares, solid gray line), or the HF diet switched to the low-fat (LF) diet (HF→LF; black squares, solid black line). Time points marked with an asterisk (*) denote significant differences in the HF→LF group compared to both the HF and HF→HFB groups (*p* < 0.05). Bars in (**B**) not sharing a lower-case letter differ (*p* < 0.05). Diet intake (**C**) and energy intake (**D**) are shown for only the eight weeks of dietary intervention in established obesity.

BW changes over the 12-week obesity development phase along with changes in BW following the subsequent eight-week dietary intervention phase within established obesity are shown in Figure 1A. Initial BW did not differ between mice at the outset of the dietary intervention or after 12 weeks of HF intake to establish the obese phenotype, as shown previously [32]. There was no difference in BW between the HF and HF→HFB groups at any time point during the eight weeks of dietary intervention (Figure 1A). Conversely, HF→LF diet-fed mice started losing weight after two weeks of LF diet consumption, and a significant reduction in BW compared to the HF and HF→HFB groups was apparent between weeks 15 and 20 (Figure 1A). Similarly, epididymal adipose tissue (EAT) depot weights were significantly reduced in HF→LF mice compared to HF and HF→HFB, which did not differ from each other (Figure 1B). Weekly diet intake during the eight-week intervention phase did not differ between groups (Figure 1C). Energy intake during the eight-week intervention period (shown in Figure 1D) was highest in the HF and HF→HFB groups compared to HF→LF.

**Figure 2:**
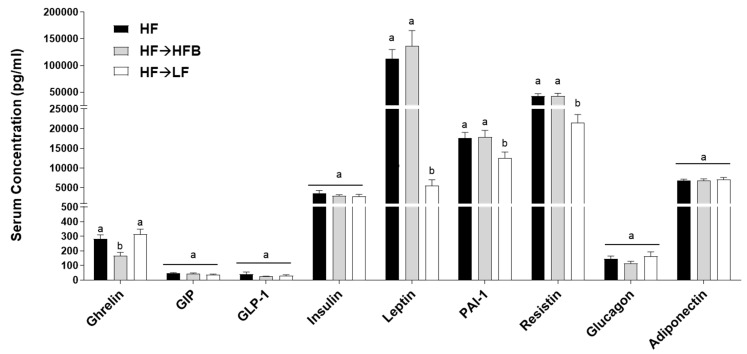
Serum hormone concentrations. Values are means ± SEM and bars not sharing a lower-case letter differ (*p* < 0.05); *n* = 12/dietary group.

Serum concentrations of obesity-associated hormones after eight weeks of dietary intervention in established obesity are shown in Figure 2. The inflammatory hormones leptin, PAI-1, and resistin were all reduced in the HF→LF group compared to both the HF and HF→HFB groups (Figure 2), indicating an improvement in the obese phenotype with the introduction of caloric restriction in established obesity. There was no difference in the serum hormone concentrations between the HF and HF→HFB groups with the exception of ghrelin, which was reduced only in the HF→HFB group compared to both the HF and HF→LF groups (Figure 2). There was no difference in the serum hormone concentrations of GIP, GLP-1, insulin, glucagon, adiponectin, and LBP (Supplementary Figure S1) between any dietary groups.

### 3.2. Changes in Intestinal Health: Microbiota Composition and Function

**Figure 3:**
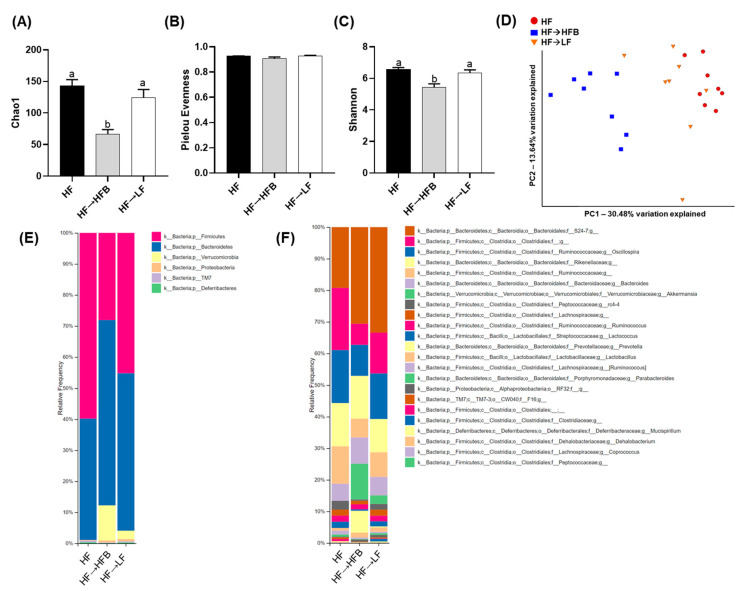
Fecal microbiota α-diversity, β-diversity, and relative taxa abundance. α-diversity metrics within treatment groups analyzed by Chao1 (**A**), Pielou’s evenness (**B**), and Shannon diversity (**C**). Bars not sharing a lower-case letter differ (*p* < 0.05). Principal component analyses (PCoA) of unweighted UniFrac distance matrices (**D**) demonstrating that the bacterial communities clustered within the diet groups and the percentage of dataset variability was PC1: 30.48% and PC2: 13.64%. Each group is represented by a color (red: HF; blue: HF→HFB; green: HF→LF), and each dot represents one mouse. Microbiota taxa composition at the phylum (**E**) or family (f)/genus (g) level (includes taxa representing >0.5% total composition in at least one sample) (**F**); *n* = 8/dietary group.

We determined the effect of the introduction of a bean supplemented HF diet within established obesity on parameters of intestinal health by first focusing on the fecal microbiota community structure as assessed by 16S rRNA gene sequencing. Differences in phylogenetic diversity within samples (α-diversity) assessed using Chao1, Pielou’s evenness, and Shannon is shown in Figure 3A–C. There was no difference in α-diversity (assessed by any approach) between the HF and HF→LF groups (*p* > 0.05); however, α-diversity was lower in the HF→HFB group compared to the HF and HF→LF groups as assessed by Chao1 and Shannon. Subsequently, β-diversity differed significantly between dietary groups (PERMANOVA: HF vs. HF→HFB, *p* = 0.0015; HF vs. HF→LF, *p* = 0.02; HF→HFB vs. HF→LF, *p* = 0.0015) and was visualized as the PCoA of the unweighted UniFrac distance matrices (Figure 3D). Supplementary Figure S2 displays PCoA plots and associated PERMANOVA *q*-values for Jaccard, Bray–Curtis, and weighted UniFrac distance matrices, which demonstrated similar differences in β-diversity between dietary groups.

The relative fecal microbial taxa abundance within groups at the phylum and genus or species levels is shown in Figure 3E,F and Table 2. A comparison of the two high-fat dietary groups, namely HF versus HF→HFB, demonstrated that the introduction of beans in established obesity, while still maintaining the intake of an obesogenic diet, altered the fecal microbiota community structure, most notably by increased relative abundance of *Akkermansia muciniphila*, whose abundance has been shown to decrease in obese rodents [48,49] and in humans living with obesity [50,51,52,53,54] and was undetectable in the HF group.

Moreover, there was increased relative abundance of the Bacteroidetes phylum in the HF→HFB group compared to the HF group, primarily due to increases in the carbohydrate-fermenting [55,56] bacteria *Prevotella* (7.81-fold), S24-7 (1.6-fold), and the Bacteroides. Conversely, the HF→HFB group had reduced relative abundance of the Firmicutes phylum, which was attributable to the reduced abundance of the Clostridiales order, the *Ruminococcaceae* family, and *Lactococcus* genus compared to HF and HF→LF groups. Additionally, the relative fecal abundance of the *rc4-4* genus was reduced in only the HF→HFB group compared to HF.

As a result of switching to a LF diet within established obesity (i.e., the HF→LF group), there were minimal effects on the composition of the microbiota. Compared to both the HF and HF→HFB groups, mice switched to the LF diet had reduced relative abundance of the *Clostridiaceae* family. Furthermore, there were changes that were common in both the HF→LF and the HF→HFB groups compared to HF alone, namely increased abundance of the *S24-7* family and reduced abundance of the *Ruminococcaceae* family.

**Figure 4:**
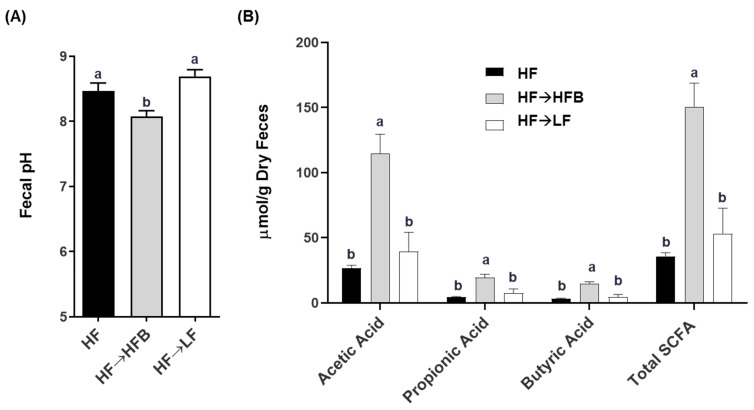
The effect of diet on fecal pH (**A**) and short-chain fatty acid (SCFA) concentration (**B**). Bars not sharing a lower-case letter differ (*p* < 0.05). Values are means ± SEM; *n* = 12/dietary group.

Fecal pH was reduced in the HF→HFB-fed mice compared to both the HF and HF→LF mice, which did not differ from each other (*p* > 0.05; Figure 4A). Furthermore, concentrations of fecal SCFAs (total and acetic acid, propionic acid, and butyric acid) were increased in the HF→HFB mice compared to HF and HF→LF mice (Figure 4B), an effect we had observed previously in lean [15,16,18] and obese mice [32] fed with bean supplemented diets.

### 3.3. Changes in Intestinal Health: Colonic Microenvironment

**Figure 5:**
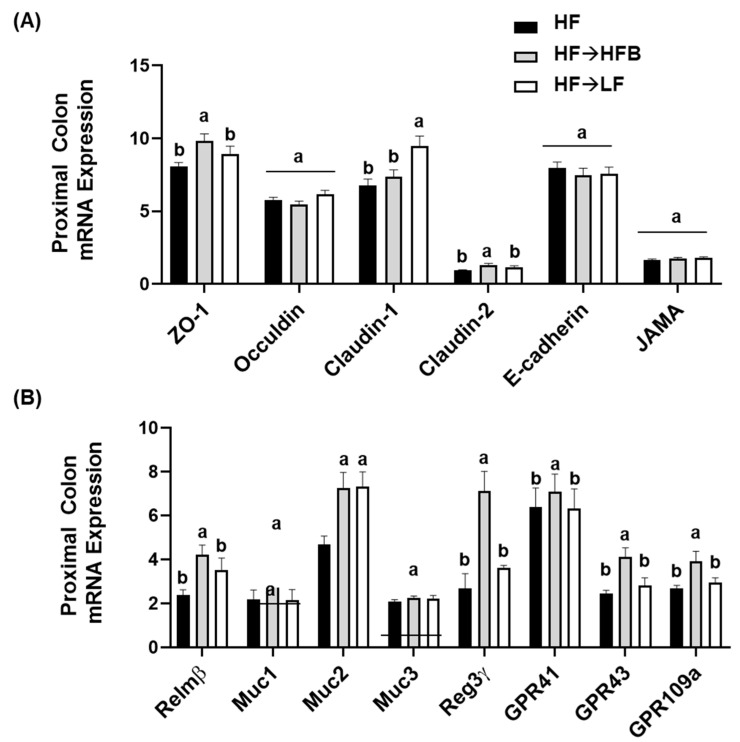
Proximal colon mRNA expression of apical junctional complex components (**A**) and markers of epithelial barrier function (**B**). Values are means ± SEM; *n* = 12/dietary group. Bars not sharing a lower-case letter differ (*p* < 0.05). Data for each gene was normalized to the expression of the housekeeping gene *RPLP0*.

Minimal changes in intestinal size parameters were influenced by diet (e.g., colon length and weight; data not shown); however, cecum weight was significantly increased in the HF→HFB (0.056 ± 0.0028 g) group compared to HF (0.045 ± 0.0021 g) and HF→LF (0.046 ± 0.0032 g) groups (*p* = 0.02). Changes in proximal colon gene expression of apical junctional complex components are shown in Figure 5A. Proximal colon mRNA expression of *ZO-1* and *claudin-2* were increased in the HF→HFB group compared to both the HF and HF→LF groups. Conversely, only claudin-1 expression was increased in the HF→LF group. There was no difference in gene expression of *occludin*, *E-cadherin*, or *JAMA* between dietary groups.

Colon mRNA expression of resistin-like molecule beta (*Relmβ*), a goblet cell-derived protein that enhances mucosal barrier integrity and mucin secretion [57], was increased in the HF→HFB group compared to both the HF and HF→LF groups (Figure 5B). Similarly, in the HF→HFB group, mRNA expression of *Muc2* was increased (Figure 5B), which is the secreted mucin responsible for protection of the epithelial barrier [57]; however, there was no difference between dietary groups in mRNA expression of the epithelial-associated mucins *Muc1* and *Muc3.* Further, regenerating islet-derived protein-3γ (*Reg3γ*), which reduces microbial–host interactions at mucosal surfaces [58,59,60], was increased only in the HF→HFB group compared to the HF and HF→LF groups (Figure 5B). Additionally, colonic mRNA expression of the G-coupled protein receptors (GPR), specifically *GPR-41*, *GPR-43*, and *GPR-109a*, for which SCFA are ligands, were all increased in the HF→HFB group compared to both the HF and HF→LF groups (Figure 5B).

### 3.4. Changes in Visceral AT Inflammation

**Figure 6:**
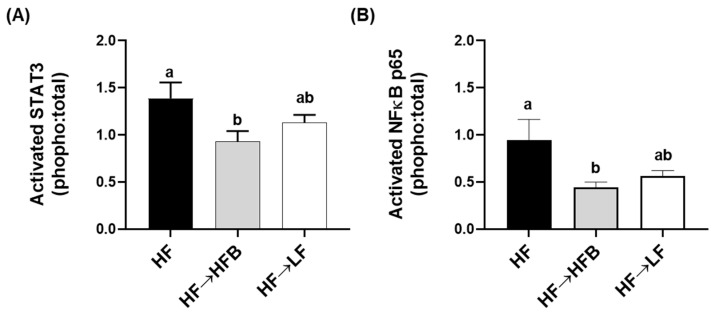
EAT NFκB p65 (**A**) and STAT3 (**B**) activation. Values are means ± SEM; *n* = 12/dietary group. Bars not sharing a lower-case letter differ (*p* < 0.05). Data are presented as the ratio of phosphorylated to total protein expression.

**Figure 7:**
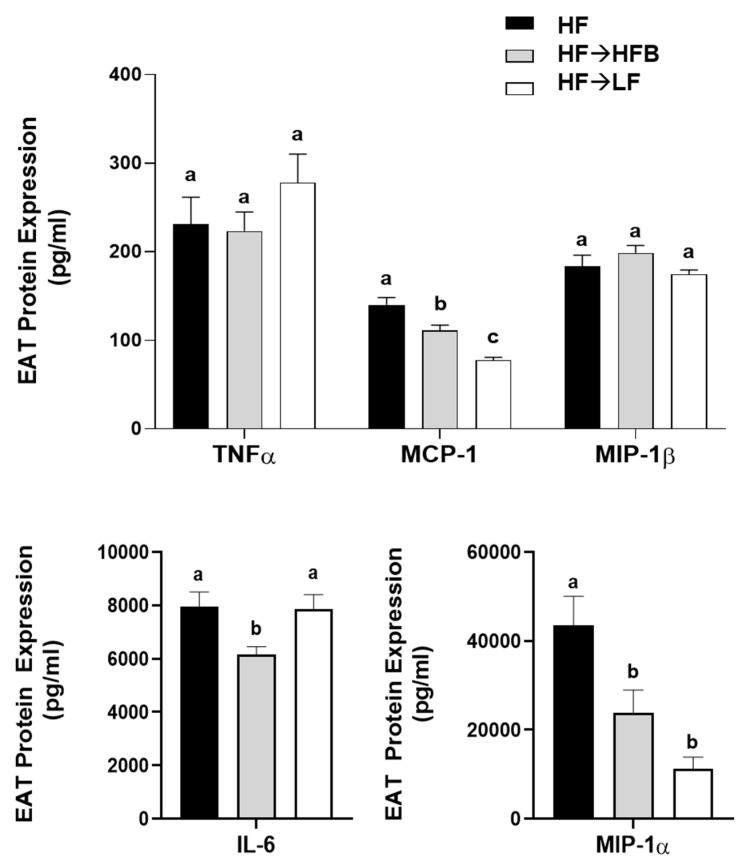
EAT cytokines and chemokine protein expression. Values are means ± SEM; *n* = 12/dietary group. Bars not sharing a lower-case letter differ (*p* < 0.05).

Epidydimal AT (EAT) activation (i.e., phosphorylated to total protein expression) of the inflammatory transcription factors STAT3 and NFκBp65 were reduced in the HF→HFB group compared to both HF alone and the HF→LF group (Figure 6), despite remaining on a HF and not exhibiting weight loss. Downstream of the transcription factors, EAT protein expression of inflammatory cytokines and chemokines were also reduced in the HF→HFB group, as shown in Figure 7. Specifically, in the HF→ HFB group, EAT protein expression of IL-6 was reduced compared to both the HF and HF→LF groups, which did not differ from each other. Expression of the inflammatory chemokines MCP-1 and MIP-1α were also reduced in the HF→HFB group compared to HF alone, and this reduction was also apparent in the HF→LF group compared to HF alone. Conversely, there was no difference in EAT protein expression of TNFα and MIP-1β between any dietary groups.

## 4. Discussion

The current study determined the effect of a cooked navy bean supplemented high-fat diet (60% kcal as fat; HF→HFB) in attenuating the severity of established obesity compared to either a weight loss control group via switching to a low-fat diet (HF→LF) or a HF control (i.e., no intervention/remaining obese). Few studies have been conducted to determine the effects of bean interventions in established obesity, and they are usually combined with caloric restriction and utilize changes in body weight and/or BMI as the primary endpoint (reviewed in [25]). Previously, we have shown the intestinal health-promoting effects of cooked beans in lean mice when supplemented into a low-fat basal diet [15,16,18]. Furthermore, we have comprehensively documented the effect of the same high-fat navy bean supplemented diet used in the current study on the obese phenotype when consumed concurrent with the development of obesity [32]. In this context, the magnitude of the resultant obese phenotype produced with bean supplementation during obesity development was attenuated, as evidenced by enhanced intestinal health (microbiome and epithelial barrier function), reduced metabolic dysfunction, and AT inflammation [32]. Although these findings highlight the potential for common beans to limit the negative impacts of a HF when consumed during the development of obesity, the translational potential of this model in humans is limited to habitual bean consumption concurrent with obesity development. Conversely, the model utilized in the current study likely exhibits greater translational potential by determining the effect of the introduction of beans as a dietary intervention in established obesity while still maintaining the intake of an obesogenic high-fat diet. Importantly, the level of bean supplementation utilized in the current study mimics the intake level of Canadian pulse consumers [29], further emphasizing the translation potential of these findings. Thus, in the HF→HFB group, despite no change in body weight (Figure 1), the introduction of a bean supplemented high-fat diet attenuated AT inflammation (reduced protein expression of NFκBp65, STAT3, IL-6, MCP-1, and MIP-1α; Figure 6 and Figure 7) and improved intestinal health, as evidenced by changes in the fecal microbiota composition (higher relative abundance of *Akkermansia muciniphila* and *Prevotella* and *S24-7*; Table 2 and Figure 3), increased SCFA concentrations (Figure 4), and increased mRNA expression of markers of intestinal function, including apical junctional complex components (*ZO-1* and *claudin-2*), regulators of mucin secretion and mucins (*Relmβ* and *Muc2*), and antimicrobial defense (*Reg3γ*) (Figure 5).

Conversely, the low-fat weight loss control group (HF→LF) exhibited a different impact on the established obese phenotype, resulting in a reduction in body weight (Figure 1) and circulating concentrations of inflammatory hormones, such as leptin, resistin, and PAI-1, versus the HF and HF→HFB groups (Figure 2); however, there were minimal improvements in intestinal health compared to HF. Specifically, switching to a low-fat diet (HF→LF) had a modest or no effect on the diversity, structure, and function of the microbiota compared to the HF (Table 2 and Figure 3 and Figure 4), and only colon mRNA expression of *claudin-1* was increased compared to HF (Figure 5). Moreover, the inflammatory profile of AT from the HF→LF group showed some improvements; however, only the macrophage chemotaxins, MCP-1, and MIP-1α were significantly reduced when compared to the HF group. Therefore, beneficial outcomes were observed in the weight loss control group (HF→LF). However, data from the current study identified a broad range of beneficial changes in intestinal health and AT function with the introduction of beans to a high-fat diet within established obesity.

The HF→HFB group did not demonstrate any weight loss, which is not unexpected given that they continued to consume a high-fat bean supplemented diet that was isocaloric with the HF diet, whereas the HF→LF weight loss control group did lose weight compared to the HF and HF→HFB groups (Figure 1). Serum ghrelin levels were reduced in the HF→HFB group; therefore, further study into the impact of bean supplementation on food intake is required. Observational studies have shown that short-term pulse consumption combined with energy restriction in overweight and obese individuals is a more effective weight loss strategy versus energy restriction alone (reviewed in [25]). Moreover, a meta-analysis showed that short-term consumption of pulses combined with caloric restriction was associated with a loss of 1.74 kg, whereas pulse consumption combined with a weight maintenance diet was associated with only a loss of 0.29 kg of body weight [26]. Therefore, if the primary outcome to designate a successful dietary intervention in obesity is achievement of weight loss, pulses (including beans) are not likely to elicit a substantial impact on this outcome. Obesity, however, is a complex condition comprising dysfunction within the intestinal microenvironment, namely microbial dysbiosis [61,62,63,64,65,66] and intestinal barrier dysfunction [5,6,67], which contribute to the development of increased adiposity, AT and systemic inflammation, and insulin resistance [2,67,68,69,70,71]. Utilizing a dietary approach to improve intestinal health (microbiota community structure and host epithelial barrier integrity) and/or host AT dysfunction may represent a more meaningful impact on the overall obese phenotype, which can be overlooked if dietary interventions are only centered on body weight or circulating mediators. Therefore, preclinical studies using animal models, such as the current study, can be utilized to identify appropriate outcomes to be assessed in human studies, particularly the human intestinal microenvironment [8,72,73,74].

The potential for cooked bean supplemented diets to promote intestinal health have been established in lean [15,16,18] and obese [30,32] mice. In the current study, we demonstrated a reproducible effect of navy bean supplementation to an obesogenic diet on intestinal health parameters, which is now apparent in two distinct obesity models, namely navy bean supplementation during the development of obesity [32] and the introduction of navy beans within established obesity. Specifically, consistent changes in the fecal microbiota community structure were reproduced in the current study, highlighting the impact of bean consumption on the composition of the obese microbiota and the utility of this type of dietary intervention to modulate obesity-associated dysbiosis regardless of when beans are introduced (i.e., concurrent with obesity development or intervention in an established obese phenotype). These changes most notably include the increased abundance of *Akkermansia muciniphila*, the abundance of which has been shown to be reduced in obese rodents [48,49] and in individuals living with obesity [50,51,52,53,54], as well as the increased abundance of *Prevotella* and *S24-7*, bacterial taxa that have been shown to enhance carbohydrate fermentation and SCFA production [55,56]. Further, these changes are concurrent with increased SCFA production and reduced fecal pH (Figure 4 and [32]). Other reproducible changes in the fecal microbiota community structure of mice consuming HFB ([32] and the current study) include decreased abundance of the *Ruminococcaceae* family and the *Lactococcus* and *rc4-4* genera. Moreover, consistent changes in host intestinal health in response to the high-fat navy bean supplemented diet (Figure 5 and [32]) include increased colon mRNA expression of apical junctional complex components; *ZO-1* and *claudin-2,* which contribute to the maintenance of epithelial barrier integrity [75]; and *Reg3γ*, the antimicrobial protein that functions to maintain a physical separation between the microbiota and the host tissues by limiting bacterial colonization of mucosal surfaces [58]. In addition to increased expression of *Muc2,* the HF→HFB group exhibited increased colon mRNA expression of *Relmβ*, which functions to promote mucosal barrier integrity by upregulating mucin secretion [57]. Similar changes in gene expression of mucins, antimicrobial proteins, and apical junctional complex components have been observed in lean mice consuming cooked bean powder supplemented diets in both healthy unchallenged and experimentally induced colitis models [15,16,18]. Although serum LBP levels did not differ between dietary groups (Figure S1), there are other serum and fecal markers reflective of barrier permeability that should be assessed in future studies [76]. Collectively, these data highlight the reproducible impact of bean supplementation on intestinal health.

Beans were supplemented into the diet as a whole food; therefore, we cannot definitively attribute the effects observed in the current study to any one component. However, the improvements in the obese phenotype were most likely attributable to the production of SCFAs. For instance, the HF→HFB group displayed elevated SCFA concentrations (Figure 4) and colonic mRNA expression of *GPR41*, *GPR43*, and *GPR109a* (Figure 5), which mediate SCFA signaling [77]. Specifically, butyric acid (butyrate) supports commensal bacterial [78] and intestinal epithelial cell growth [79,80,81], enhances mucosal barrier function and integrity by stimulating goblet cell mucus secretion [82] and epithelial tight junction protein expression [83], and suppresses the activation of inflammatory signaling pathways in humans and animal models of inflammatory bowel disease [79,80,84,85,86,87,88]. However, we cannot overlook the potential contribution of navy bean phenolic compounds to the outcomes reported herein, which are retained in cooked bean powders [89], can promote intestinal mucosal barrier function [90,91], and alter the composition and function of the intestinal microbiota [92,93,94,95]. Therefore, further research is needed to ascertain the specific effects of bean-derived bioactives on the obese phenotype.

Extra-intestinally, AT activation of inflammatory transcription factors (NFκB p65 and STAT3) and expression of inflammatory cytokines (IL-6) and chemokines (MCP-1 and MIP-1α; Figure 6 and Figure 7) were reduced in the HF→HFB group, indicative of reduced obese AT inflammatory status. Navy bean supplementation concurrent with high-fat diet-induced obesity development demonstrated similar effects on inflammatory transcription factor activation and AT gene expression [32]. This reproducible anti-inflammatory effect is likely attributable to SCFA, wherein AT mRNA expression of the SCFA signaling receptors (GPR-41, -43, and -109a) were increased in bean supplemented high-fat diet-fed mice [32]. Moreover, SCFA-stimulated adipocytes exhibit reduced lipid uptake and adipocyte differentiation as well as increased expression of peroxisome proliferator-activated receptor (PPAR)-δ and mitochondrial uncoupling protein-2, which promote energy expenditure [96]. Furthermore, SCFA supplementation to an obesogenic diet has been shown to reduce adipocyte size [97], visceral AT mass [98], and promote the beiging of adipocytes [99]. Collectively, although circulating SCFA concentrations were not measured in the current study, the anti-inflammatory effect induced by the HFB diet in the AT may be a result of SCFA signaling; however, further investigation is required.

## 5. Conclusions

The results from the current study highlight the effect of introducing beans into an established obese phenotype while maintaining intake of the obesogenic diet. Importantly, in both our previous work, determining the effect of beans during the development of obesity [32], and the current study, determining the effect of a bean supplemented diet in established obesity, typical bean preparation methods were recapitulated by preparing beans using common cooking practices (soaking and slow-cooking), and we used a bean supplementation level that corresponds to an achievable intake level in humans [29,35,36,37], which overall increases the translational potential of this study. Furthermore, cooked bean powders can be considered a “value-added” ingredient through which bean intake levels can be enhanced to promote health. This supplementation strategy has been used successfully in a recent cohort of colon cancer survivors and children at risk of CVD, in which participants consumed dishes supplemented with a cooked navy bean powder for four weeks, resulting in improved microbial diversity, composition, and metabolite profile [100,101] and increased anti-inflammatory and cardio-protective metabolites [102]. In our current study, multiple components of the established obese phenotype were improved by beans, including the intestinal microbiota composition, which included an increased relative abundance of *Akkermansia muciniphila*, whose reintroduction by daily gavage in obese rodents has been shown to improve multiple aspects of the obese phenotype [103] and whose probiotic administration to individuals living with obesity/overweight resulted in improved metabolic parameters [104]. The current study demonstrates the restoration of *Akkermansia muciniphila* within the community structure with a bean dietary intervention. Additionally, bean supplementation increased carbohydrate fermentation and the production of SCFA, increased mRNA expression of host epithelial barrier function and markers of intestinal health and reduced AT inflammation. Collectively, the current investigation adds to and supports our previous findings demonstrating a consistent effect of cooked beans to beneficially enhance intestinal health in healthy unchallenged mice and attenuate intestinal dysfunction and microbial dysbiosis in mouse models of obesity and experimental colitis [15,16,18,32].

## Figures and Tables

**Table 1:** Diet composition.

Ingredients (g/kg)	High-Fat (HF)	High-Fat + Bean (HF→HFB)	Low-Fat (HF→LF)
Bean Powder	0.0	157.0	0.0
Casein	265.0	220.4 ^1^	265.0
L-cystine	4.0	4.0	4.0
Corn starch	0.0	0.0	297.0
Maltodextrin	155.5	155.5	155.5
Sucrose	90.0	50.5 ^1^	90.0
Lard	234.0	234.0	19.0
Corn oil	106.0	103.1 ^1^	24.0
Cellulose	70.0	0.0 ^1^	70.0
Mineral mix (AIN-93G-MX)	48.0	48.0	48.0
Calcium phosphate, dibasic	3.4	3.4	3.4
Vitamin mix (AIN-93G-VX)	21.0	21.0	21.0
Choline bitartrate	3.0	3.0	3.0
Tertiary butylhydroquinone (TBHQ)	0.02	0.02	0.005
Energy density (kcal/kg)	5237	5223	3633

^1^ To equalize protein, available carbohydrates, lipid, and fiber content (g/kg diet) between the HF and HF→HFB diets, casein, sucrose, corn oil, and cellulose were reduced in the HF→HFB diet, respectively, to account for the protein, available carbohydrate, fat, and total fiber present in the navy bean powder as determined by Maxxam Analytics proximate analysis: ash 4.4%, fat 1.83%, protein 25.3%, available carbohydrate 23.1%, moisture 0.9%, total dietary fiber 44.5%, and soluble fiber 5.3%.

**Table 2:** Fecal microbial relative taxa abundance in mice fed with HF, HF→HFB, and HF→LF diets.

Taxonomy	HF	HF→HFB	HF→LF
Bacteroidetes	38.38 ± 4.79	61.73 ± 3.13 *	48.75 ± 3.74 ^#^
Bacteroidetes;f__Rikenellaceae	13.43 ± 2.18	14.11 ± 1.33	10.54 ± 0.75
Bacteroidetes;f__S24-7	18.89 ± 2.59	30.37 ± 1.08 *	31.67 ± 3.45 *
Bacteroidetes;g__Bacteroides	5.42 ± 0.38	9.16 ± 1.00 *	5.69 ± 0.43 ^#^
Bacteroidetes;g__Parabacteroides	0.64 ± 0.19	0.28 ± 0.15	0.54 ± 0.18
Bacteroidetes;g__Prevotella	0 ± 0	7.81 ± 1.47 *	0.31 ± 0.12 ^#^
Deferribacteres	0.33 ± 0.08	0.09 ± 0.09 *	0.27 ± 0.13 ^#^
Deferribacteres;g__Mucispirillum	0.33 ± 0.08	0.09 ± 0.09 *	0.27 ± 0.13 ^#^
Firmicutes	60.5 6 ± 4.82	26.9 ± 4.05 *	47.61 ± 4.44 ^#^
Firmicutes;f__Clostridiaceae	0 ± 0	0 ± 0	0.77 ± 0.20 *^#^
Firmicutes;f__Lachnospiraceae	2.00 ± 0.47	1.09 ± 0.78	2.08 ± 0.40
Firmicutes;f__Peptococcaceae	0 ± 0	0.02 ± 0.02	0.04 ± 0.02
Firmicutes;f__Ruminococcaceae	12.10 ± 0.90	5.43 ± 1.34 *	7.74 ± 0.81 *
Firmicutes;g__Coprococcus	0.11 ± 0.07	0.02 ± 0.02	0 ± 0
Firmicutes;g__Dehalobacterium	0.14 ± 0.09	0.01 ± 0.10	0.16 ± 0.09
Firmicutes;g__Lactobacillus	0.79 ± 0.33	1.43 ± 0.58	1.24 ± 0.41
Firmicutes;g__Lactococcus	2.08 ± 0.31	0.43 ± 0.22 *	1.53 ± 0.32 ^#^
Firmicutes;g__Oscillospira	16.97 ± 1.25	9.11 ± 1.75	15.95 ± 2.90
Firmicutes;g__rc4-4	2.88 ± 0.40	0.52 ± 0.26 *	1.65 ± 0.34
Firmicutes;g__Ruminococcus	1.94 ± 0.56	1.41 ± 0.43	2.24 ± 0.88
Firmicutes;g__Ruminococcus gnavus	1.30 ± 0.35	0.59 ± 0.46	0.4 ± 0.21
Firmicutes;o__Clostridiales	19.64 ± 2.75	6.75 ± 1.74 *	13.75 ± 1.36 ^#^
Proteobacteria	0.19 ± 0.07	0.57 ± 0.22	0.68 ± 0.19
Proteobacteria;o__RF32	0.19 ± 0.07	0.57 ± 0.22	0.68 ± 0.19
TM7	0.53 ± 0.17	0.14 ± 0.12	0.39 ± 0.11
TM7;f__F16	0.53 ± 0.17	0.14 ± 0.12	0.39 ± 0.11
Verrucomicrobia	0 ± 0	10.57 ± 2.57 *	2.31 ± 0.88 ^#^
Verrucomicrobia;g__Akkermansia muciniphila	0 ± 0	10.57 ± 2.57 *	2.31 ± 0.88 ^#^

Fecal microbial relative taxa abundance (mean ± SEM) at the phylum level (gray shaded rows) and corresponding family (f), genus (g), and/or species (italics). Significantly different values (*p* < 0.05; false discovery rate (FDR) < 0.05) are identified using the following symbols: (*) differs from HF and (#) differs from HF→HFB as determined by Dunn’s multiple comparison test; *n* = 8/dietary group.

## Data Availability

Data is contained within the article or supplementary material. The data presented in this study are available upon request to the corresponding author.

## Supplementary Materials

The following are available online at https://www.mdpi.com/2072-6643/13/3/757/s1, Figure S1: Serum lipopolysaccharide binding protein (LBP) concentrations in mice. Data indicate mean ± SEM. There was no difference detected between groups as analyzed by one-way ANOVA followed by Tukey’s multiple comparison test; (*n* = 10–11/group); Figure S2: Fecal microbiota β-diversity. PCoA of Jaccard distance matrices (A), Bray-Curtis distance matrices (B), and (C) Weighted Unifrac distance matrices, demonstrating that the bacterial communities clustered within diet groups; PERMANOVA q-values are shown in (D). HF: red circles, HF→HFB: blue squares, HF→LF: orange triangles.
