# Supplementary material for: Navy Bean Supplementation in Established High-Fat Diet-Induced Obesity Attenuates the Severity of the Obese Inflammatory Phenotype
Source: Nutrients. 2021 Feb 26;13(3):757. doi: 10.3390/nu13030757 (PMC7996849; doi:10.3390/nu13030757)
Supplement: Supplementary file 1 [file nutrients-13-00757-s001.pdf]

## Supplemental Materials

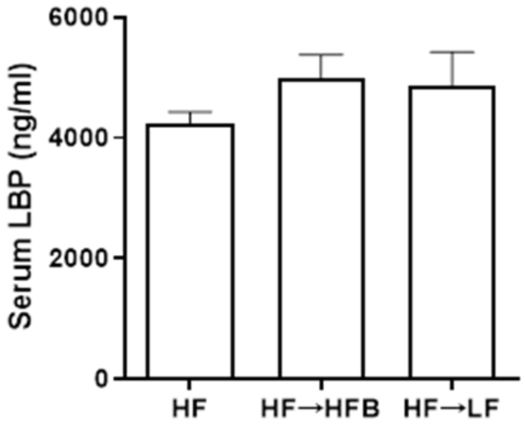


**Figure S1:** Serum lipopolysaccharide binding protein (LBP) concentrations in mice. Data indicate mean ± SEM. There was no difference detected between groups as analyzed by one-way ANOVA followed by Tukey's multiple comparison test; (n=10-11/group).

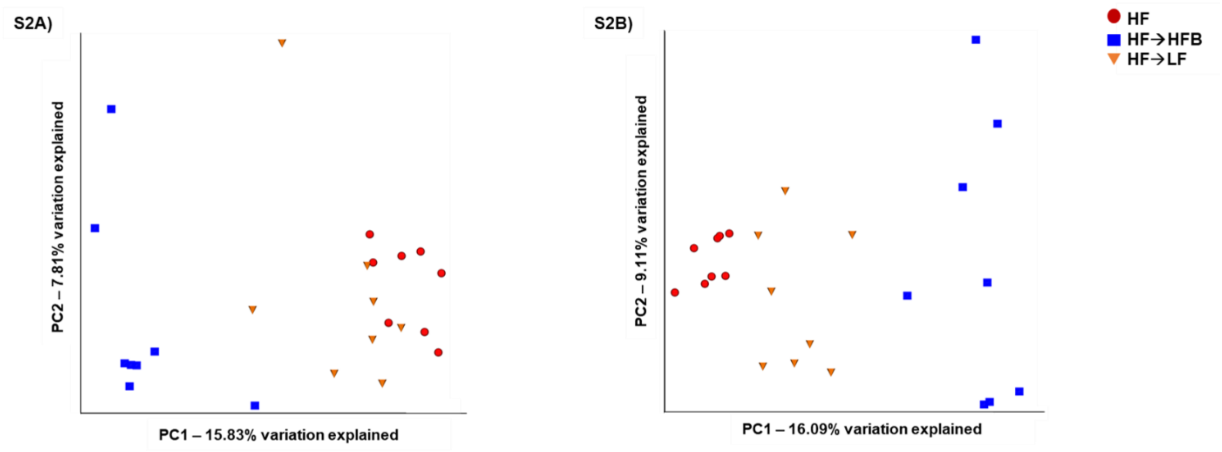


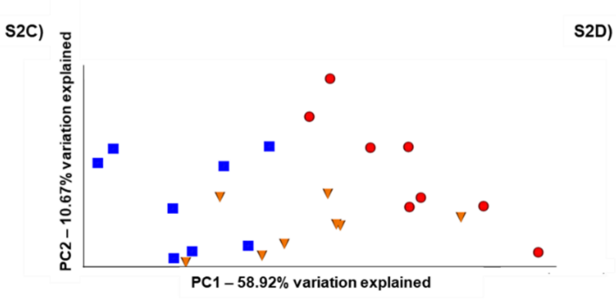


S2D)

| | PERMANOVA q-value | | |
|---|---|---|---|
| Distance matric | HF vs HF→HFB | HF vs HF→LF | HF→HFB vs HF→LF |
| Jaccard | 0.001 | 0.001 | 0.001 |
| Bray-Curtis | 0.0015 | 0.0020 | 0.0015 |
| Weighted unifrac | 0.0015 | 0.0080 | 0.0015 |

**Figure S2**. Fecal microbiota β-diversity. PCoA of Jaccard distance matrices (A), Bray-Curtis distance matrices (B), and (C) Weighted Unifrac distance matrices, demonstrating that the bacterial communities clustered within diet groups; PERMANOVA q-values are shown in (D). HF: red circles, HF→HFB: blue squares, HF→LF: orange triangles.
